# Utilization of the Naranjo scale to evaluate adverse drug reactions at a free-standing children’s hospital

**DOI:** 10.1371/journal.pone.0245368

**Published:** 2021-01-13

**Authors:** Madhavi Murali, Sarah L. Suppes, Keith Feldman, Jennifer L. Goldman

**Affiliations:** 1 School of Medicine, University of Missouri–Kansas City, Kansas City, MO, United States of America; 2 Division of Clinical Pharmacology, Children’s Mercy Kansas City, Kansas City, MO, United States of America; 3 Department of Pediatrics, Children’s Mercy Kansas City and the University of Missouri-Kansas City, Kansas City, MO, United States of America; Hospital JP Garrahan, ARGENTINA

## Abstract

The relationship between the Naranjo scaling system and pediatric adverse drug reactions (ADR) is poorly understood. We performed a retrospective review of 1,676 pediatric ADRs documented at our hospital from 2014–2018. We evaluated patient demographics, implicated medication, ADR severity, calculated Naranjo score, associated symptoms, and location within the hospital in which the ADR was documented. ADR severity was poorly correlated with Naranjo interpretation. Out of the 10 Naranjo scale questions, 4 had a response of “unknown” greater than 85% of the time. Cardiovascular and oncological/immunologic agents were more likely to have a probable or definite Naranjo interpretation compared to antimicrobials. Further strategies are needed to enhance the causality assessment of pediatric ADRs in clinical care.

## Introduction

According to the World Health Organization, an adverse drug reaction (ADR) is “a response to a medicine which is noxious and unintended, and which occurs at doses normally used in man” [1,2]. Together, the frequent use of off-label medications along with developmental changes that occur in children from infancy to adolescence result in an elevated risk for ADRs [3,4]. Despite the known ADR risk, pediatric ADRs often are underrecognized and underreported [4,5]. Moreover, even when ADRs are documented in the electronic medical record (EMR), the information often lacks the details required for medical providers to determine how to safely prescribe medications related to the implicated drug. Effectively doing so requires the determination of key factors such as ADR phenotype and severity, as many ADRs are tolerable side effects with low risk of recurrence while others can be severe and life threatening [6]. Yet, without available clinical tests to confirm or negate if a drug caused a reaction, the medical provider is challenged on how to interpret a potential ADR.

As a result, causality assessment tools (CATs) have been developed to provide an objective, standardized approach to aid in the determination of whether or not an administered drug caused an ADR [7,8]. CATs are typically comprised of questions focused on several themes including the drug exposure, adverse reaction presentation, and patient characteristics. Unfortunately, the results of CATs have been shown to be highly variable, depending on the tool used and the provider using the tool [9–11]. Moreover, as the majority of causality tools have been developed for adult populations, the utility in hospitalized children remains poorly understood [12].

Among such toolsets, the Naranjo scaling system is one of the most widely utilized CATs [8]. In the case of an adverse reaction, a Naranjo assessment can be completed. This assessment is comprised of 10 questions concerning the implicated medication and reaction phenotype. Each answered question has an individual score, which is then totaled to provide a final score that is associated with one of four categories of likelihood that the drug was associated with the reaction (unlikely, possibly, probably, or definitely). To date, it is unclear if and how Naranjo results are associated with pediatric ADR clinical factors.

Accordingly, the primary objective of this work was to understand how the Naranjo is used in the pediatric setting. To do so, we characterized Naranjo probability scores with respect to pediatric ADR phenotype, severity, and implicated drug class. We then took this one step further, in a secondary objective to identify specific clinical factors associated with probable or definite Naranjo scores.

## Methods

### Study setting

We performed a retrospective case series study of hospitalized patients with a documented ADR at Children’s Mercy Kansas City, a 354-bed free-standing academic pediatric hospital with an average of 15,000 admissions annually. In October 2010, an active hospital-wide pharmacovigilance program was started to identify and evaluate children hospitalized with an active or historical ADR. Details of this program have been described in previous works [6,13,14].

ADRs are classified and documented in the electronic medical record (EMR) by phenotype (hypersensitivity, side effect, other) and severity (mild, moderate, severe) (Table 1) [6]. Severity classification are based on the Hartwig’s Severity Assessment Tool [15] with the modification that we consider a reaction moderate if the implicated drug was discontinued [6]. Clinical pharmacists who have undergone pharmacovigilance training, also collect more detailed information including patient demographics, ADR phenotype, ADR treatment, completion of the Naranjo scale, and whether the reaction occurred within 30 days Naranjo assessment. All ADR information is documented in a form within the EMR. The Naranjo scale is a series of 10 questions. Each question can be answered as “Yes”, “No”, or “Unknown”, with point values assigned to each answer [8]. The final score interpretations are stratified into four categories with a score of ≥ 9 considered “definite”, 5 to 8 “probable”, 1 to 4 “possible”, and those ≤0 “doubtful” likelihood of the drug causing the ADR.

**Table 1:** Classification of phenotype and severity of adverse drug reactions [6].

Phenotypes	Definition
Allergy/Hypersensitivity	Immune response or other mechanism unique to the patient (rash, hives, anaphylaxis)
Side effect	Undesirable response due to pharmacologic properties of drug
**Severity**	**Definition**
Mild	Drug can be continued without any treatment
Moderate	Drug was stopped and/or required treatment
Severe	Reaction caused hospital admission, permanent disability, delayed discharge, or was life threatening

### Study cohort and data collection

The data utilized for this study were obtained from the pharmacovigilance database between November 15, 2014 and December 31, 2018. From this complete set of documented and pharmacist reviewed ADRs, a series of exclusion criteria were applied to ensure a robust data set for analysis.

To begin, all children ≥21 years of age were removed. Next, all children with multiple ADRs or those with more than a single implicated drug for any reaction were excluded. Such a scenario can occur in two ways. First, there may be multiple drugs implicated for a single ADR, for which a Naranjo questionnaire would have been completed for each drug. Second, a child may have two or more ADRs documented in their record. In both cases, these data may be biased by extrinsic factors (such as time between reactions), and as such all children with multiple ADR/Naranjo records were excluded from this study. Finally, we ensured a complete record of ADR assessment by the pharmacovigilance program was available, requiring the calculated Naranjo score (and response to each Naranjo question), ADR phenotype, ADR mediation, as well as a complete set of patient demographics. The implicated drugs were further classified into general drug classes as defined by Shehab et al [16]. This study was approved by the local institutional review board.

### Statistical analysis

Descriptive statistics were computed for cohort demographics (age at time of ADR documentation, sex, race/ethnicity), as well as the clinical characteristics of the documented ADR (reaction phenotype, clinical symptoms, and overall Naranjo score). Median and interquartile range (IQR) are reported for all continuous features, and frequency distributions are used for all nominal categories.

First, to evaluate the association between Naranjo scoring and ADR attributes, a series of bivariate statistical comparisons were performed with implicated drug class, phenotype, and severity. Given the low prevalence in the highest and lowest Naranjo categorization, non-parametric Fisher’s exact tests were performed to assess the distribution of the 4 Naranjo classes across each of the study variables. Due to the large number of *drug class* categories, the Fisher’s test was performed with a Monte Carlo simulation, using 10,000 sampling iterations and seeded for reproducibility [17]. Directionality and magnitude of the relationship between ADR severity and Naranjo categories were assessed by the Kendall rank correlation coefficient (tau). In all cases, exact p-values are reported. Results that reach significance at or above 95% confidence are highlighted in the results and discussion sections to follow.

Next with respect to the secondary objective, the identification of clinical factors associated with the Naranjo categorizations (probable / definite vs. possible / doubtful scores), multivariate logistic regression was performed adjusting for study variables found to be significant in the bivariate analyses: drug class, severity, and phenotype. In addition, regressions were adjusted for patient age, sex, and race/ethnicity. Reference categories were selected to represent the lowest severity level, largest drug class and race/ethnicity.

Finally, in an effort to better contextualize the data presented throughout this manuscript, we concluded with an analysis focused on the Naranjo questionnaire itself, where the frequency of “yes”, “no”, and “unknown” responses were calculated across each Naranjo score question. All analyses used python 3.7.6, pandas 1.0.1, numpy 1.18, scipy 1.4.1, and statsmodels 0.11.

## Results

In total, 1746 children with a single ADR were identified by the pharmacovigilance program during the study period. Of these, 70 ADRs failed to meet the remaining inclusion criteria: starting with patients older than 21 years of age (n = 6), those with missing Naranjo scores (n = 31), missing severity classification (n = 14), or failure to document if the reaction occurred within past 30 days (n = 7), classifications other than hypersensitivity reactions or side effects (n = 2), missing and unknown race (n = 10), resulting in a total of 1676 ADRs included in analysis.

Of the 1,676 ADRs, the median age of a patient with a documented ADR was 10.5 years (IQR, 5–15), 822 (49%) of patients were male, and the majority of patients were Caucasian 1,286 (77%) (Table 2). Hypersensitivity reactions were documented 66% of the time as compared to side effects (34%). The most common ADR clinical symptoms were cutaneous 1197 (71%), neurological 228 (14%), and gastrointestinal 256 (15%). The Naranjo score classified 25 (1.5%) of cases as definite, 835 (50%) probable, 813 (49%) possible, and 3 (0.2%) doubtful (Table 2). The adverse reaction severity as classified by a pharmacist totaled 25 (1.5%) mild, 1489 (89%) moderate, and 162 (10%) severe reactions (Table 3).

**Table 2:** Characteristics of pediatric patients with an ADR (N = 1676).

Clinical Characteristics at the time of ADR documentation	
Age, y, median (IQR)	10.5 (5–15)
Male, N (%)	822 (49)
Race or ethnicity, N (%)	
Caucasian	1286 (77)
African American	131 (7.8)
Multiracial	102 (6)
Hispanic	112 (6.7)
Other	30 (1.8)
Asian	15 (0.9)
**Final reaction phenotype classified, N (%)**	
Hypersensitivity	1108 (66)
Side effect	568 (34)
**Most common ADR clinical symptoms, N (%)**	
Cutaneous	1197 (71)
Neurological	228 (14)
Gastrointestinal	256 (15)
Respiratory	174 (10)
Cardiovascular	62 (3.7)
**Naranjo score, N (%)**	
Definite (≥9)	25 (1.5)
Probable (5–8)	835 (49.8)
Possible (1–4)	813 (48.5)
Doubtful (0)	3 (0.2)

ADR, adverse drug reaction; IQR, interquartile range.

**Table 3:** Adverse reaction severity by Naranjo score interpretation.

Severity	N (%)	Doubtful (%)	Possible (%)	Probable (%)	Definite (%)
Mild	25 (1.5)	0 (0)	14 (56)	10 (40)	1 (4)
Moderate	1489 (88.8)	3 (0.2)	728 (48.9)	736 (49.4)	22 (1.5)
Severe	162 (9.7)	0 (0)	71 (43.8)	89 (55)	2 (1.2)

ADR phenotype was found to be significantly different across the Naranjo interpretation categories (P < .001). A Fisher’s test showed no statistical difference between the 3 severity levels and interpretation (P = 0.459), and upon further evaluation the correlation between increasing severity and Naranjo score was extremely low (Tau: 0.0329, P: 0.1726).

With respect to drug class, the most common implicated drugs were systemic antimicrobial agents (1072; 64%) (Table 4). Monte Carlo simulation demonstrated the distribution of Naranjo scores was not uniform across drug classifications (*P* < .001). Cardiovascular agents had the highest percentage of a definite Naranjo score interpretation (6.3%), followed by hormone-modifying agents (5.9%). When controlling for age, gender, race, time of documentation, as well as adverse reaction severity and phenotype, cardiovascular and oncological/immunologic agents were more likely to have a probable or definite Naranjo interpretation compared to the reference category of antimicrobials (Table 5). Documented ADRs that were evaluated within 30 days of occurring were also more likely to be probable or definite.

**Table 4:** General drug classification by Naranjo score interpretation.

Drug class	N (%)	Doubtful (%)	Possible (%)	Probable (%)	Definite (%)
Systemic antimicrobial agents	1072 (64)	0 (0.0)	549 (51.2)	506 (47.2)	17 (1.6)
CNS agents	284 (16.9)	1 (0.4)	124 (43.7)	155 (54.6)	4 (1.4)
Respiratory agents	76 (4.5)	0 (0.0)	43 (56.6)	32 (42.1)	1 (1.3)
Other drug classes	68 (4.1)	2 (2.9)	27 (39.7)	39 (57.4)	0 (0.0)
Oncological and immunologic agents	63 (3.8)	0 (0.0)	18 (28.6)	44 (69.8)	1 (1.6)
Musculoskeletal agents	36 (2.1)	0 (0.0)	22 (61)	14 (39)	0 (0.0)
Gastrointestinal agents	26 (1.6)	0 (0.0)	11 (42.3)	15 (57.7)	0 (0.0)
Hormone-modifying agents	17 (1)	0 (0.0)	8 (47)	8 (47)	1 (5.9)
Cardiovascular agents	16 (0.95)	0 (0.0)	4 (25)	11 (68.8)	1 (6.3)
Dietary supplements and related products	12 (0.7)	0 (0.0)	5 (41.7)	7 (58.3)	0 (0.0)
Hematologic agents	6 (0.4)	0 (0.0)	2 (33.3)	4 (66.7)	0 (0.0)

**Table 5:** Multivariate analysis of clinical factor associations with probable/definite vs. possible/ doubtful Naranjo scores.

		Odds ratio (95% CI)	p-value
	Intercept	0.60 (0.26–1.40)	0.241
**Race**	White	Reference	
	African American	1.07 (0.74–1.55)	0.730
	Asian	1.43 (0.50–4.08)	0.507
	Hispanic	0.78 (0.52–1.16	0.213
	Multiracial	0.75 (0.49–1.14)	0.179
	Other-Unknown	0.94 (0.45–1.96)	0.865
**Reaction documented within 30 days**	No	Reference	
	Yes	1.47 (1.14–1.89)	0.003
**Sex**	Male	0.97 (0.80–1.19)	0.800
**Drug class**	Antimicrobial agents	Reference	
	Hematologic Agents	1.88 (0.34–10.44)	0.473
	Hormone-modifying agents	1.20 (0.45–3.19)	0.721
	CNS agents	1.29 (0.95–1.75)	0.105
	Cardiovascular agents	3.22 (1.02–10.23)	0.047
	Oncological and immunologic agents	2.39 (1.32–4.34)	0.004
	Musculoskeletal agents	0.66 (0.33–1.33)	0.245
	Respiratory agents	0.76 (0.47–1.23)	0.262
	Gastrointestinal agents	1.41 (0.63–3.13)	0.402
	Other drug classes	1.38 (0.84–2.27)	0.207
	Dietary supplements and related products	1.51 (0.47–4.90)	0.491
**Final severity**	Mild	Reference	
	Moderate	1.28 (0.56–2.92)	0.550
	Severe	1.13 (0.47–2.74)	0.781
**Final phenotype**	Hypersensitivity	Reference	
	Side effect	0.93 (0.73–1.19)	0.589
**Age at time of ADR documentation**		1.02 (1.00–1.04)	0.020

Evaluation of each Naranjo question revealed an “unknown” response was frequently selected (Table 6). Specifically, questions regarding drug re-administration (question 4), administration of a placebo (question 6), detection of drug at toxic concentrations (question 7), and reaction in response to changes in dosing (question 8) had a response rate of “unknown” greater than 85% of the time.

**Table 6:** Frequency of responses to individual Naranjo questions.

Question	Yes N (%)	No N (%)	Unknown N (%)
1) Are there previous conclusive reports of this reaction?	1579 (94.2)	35 (2.1)	62 (3.7)
2) Did the adverse event appear after the drug was given?	1670 (99.6)	1 (0.1)	5 (0.3)
3) Did the adverse reaction improve when the drug was discontinued or a specific antagonist was given?	1607 (95.9)	14 (0.8)	55 (3.3)
4) Did the adverse reaction reappear upon re-administering the drug?	170 (10.1)	27 (1.6)	1479 (88.2)
5) Were there other possible causes for the reaction?	589 (35.1)	261 (15.6)	826 (49.3)
6) Did the adverse reaction reappear upon administration of placebo?	0 (0.00)	20 (1.2)	1656 (98.8)
7) Was the drug detected in the blood or other fluids in toxic concentrations?	4 (0.2)	102 (6.1)	1570 (93.7)
8) Was the reaction worsened upon increasing the dose? Or, was the reaction lessened upon decreasing the dose?	34 (2.0)	20 (1.2)	1622 (96.8)
9) Did the patient have a similar reaction to the drug or a related agent in the past?	134 (8.0)	839 (50.1)	703 (41.9)
10) Was the adverse event confirmed by any other objective evidence?	1171 (69.9)	234 (14.0)	271 (16.2)

## Discussion

Determining whether there is a causal relationship between an undesired reaction and a drug is an important component of pharmacovigilance [12]. As diagnostic tests are not readily available to implicate a drug, CATs are used to assess the probability of a drug resulting in an ADR [18]. In this study, we found that 1) the Naranjo scoring system is not associated with ADR severity, 2) specific medication classes implicated in ADRs had increased likelihood of receiving higher Naranjo scores and 3) several questions in the Naranjo scale are answered unknown most of the time.

A probable or definite Naranjo score was not associated with a specific ADR severity or reaction phenotype. Our findings align with previous studies demonstrating that CAT results are often categorized as “possible,” which is difficult to interpret clinically [19,20]. The classification of “possible” suggests there may be another equally likely explanation for the event and/or there is uncertainty or lack of information [21]. The purpose of this work was to explore the relationship between ADR severity and causality because both are relevant clinically. When clinicians are making prescribing decisions for those children with an ADR, the clinician is typically assessing both the likelihood a medication caused the ADR and the severity of the reaction. Clinically, the utility of the Naranjo is limited, as a life-threatening reaction associated with a drug will likely be presumed too high risk to consider re-introduction regardless of probability score classification. Similarly, an ADR of mild severity classified as “definite” would not preclude use in the future. These findings further support limitations noted of utility of the Naranjo scoring system especially as related to clinical decision making [22]. Efforts to incorporate additional data and machine learning into causality assessment are underway, though not readily available for clinical pharmacovigilance.

We found that cardiovascular agents and oncological/immunologic agents had a higher likelihood of having a probable or definite Naranjo score relative to antimicrobials. Although the reason for this is unclear, our findings do suggest that different drug classes may require specific evaluations to better understand assessment. Antimicrobial agents are commonly implicated in pediatric ADRs though a reaction such as a rash can easily be confused with a viral exanthem making causality assessment challenging [23]. Studies have demonstrated antimicrobial re-challenge often reveals the drug is well-tolerated allowing for the removal of the ADR label [24,25]. Individualized causality assessment specific to a drug class may provide clinicians more helpful information for future prescribing.

The ability for a clinical pharmacist to answer the individual Naranjo questions on reported pediatric ADRs was challenging as demonstrated by the high percentage (>85%) of answers to 4 of the questions in the Naranjo questionnaire being “unknown”. The questions routinely answered “unknown” are likely the most important to determine causality. Drug challenges that would most likely confirm causality such as ADR recurrence with re-administration of the drug or with dose escalation rarely occur. In contrast, a question regarding whether or not the adverse event appeared after the drug was given was answered yes almost universally. Both types of questions are of limited use in deciphering ADR causality. These findings suggest that regardless of ADR severity, the data needed to help determine if the reaction was caused by the drug are challenging to gather by the medical provider. Previous work utilizing a modified Naranjo assessment that excludes questions 6 through 10 has been shown to effectively categorize ADRs with a high likelihood [26]. However, such studies have yet to be conducted within the pediatric population. Information specific to children could include the number of years that have elapsed since ADR occurrence as some reactions such as penicillin allergies diminish over time [27]. Also, concomitant infectious testing results would be of interest as children have viral infections that can mimic or trigger an ADR [28]. Several other CATs have been developed to enhance causality assessment, though to date, not a single tool is universally accepted or demonstrates consistent results [7,9,11].

Limitations of this study include that it is a retrospective chart review of patients at a single institution; future multi-institutional studies can help providers better understand the variance of ADR documentation practices across institutions as well as gain a broader understanding of the clinical application of causality assessment tools in pediatric populations. Based on the need to collect ADR specific data, our findings may only be applicable to institutions with pharmacovigilance programs. Additionally, results may differ at other institutions based on local pharmacovigilance practices. Finally, causality assessments performed by clinical pharmacists and Naranjo scores can vary depending on evaluator.

## Conclusion

Establishing the association between drug exposure and an ADR is challenging. The ability to determine whether a drug did cause a reaction is important when making future medication prescribing decisions. The utility of the Naranjo scale in pediatric ADR causality is limited as the scores do not differentiate ADR severity and the responses to several of the Naranjo scale questions are “unknown” when completed by clinical pharmacists. Active pharmacovigilance programs are vital to accurately identifying and assessing ADRs in the clinical setting within pediatrics. The development of a pediatric specific ADR assessment tool that can account for drug class, reaction severity and additional clinical tests may enhance ADR causality assessment and clinical care.

## Supporting information

S1 File (XLSX)
